# Enhancing Clinicians’ Use of Electronic Patient-Reported Outcome Measures in Outpatient Care: Mixed Methods Study

**DOI:** 10.2196/60306

**Published:** 2024-10-18

**Authors:** Veerle van Engen, Igna Bonfrer, Kees Ahaus, Monique Den Hollander-Ardon, Ingrid Peters, Martina Buljac-Samardzic

**Affiliations:** 1 Erasmus School of Health Policy & Management Erasmus University Rotterdam Rotterdam Netherlands; 2 Department of Quality and Patient Care Erasmus Medical Center Rotterdam Netherlands

**Keywords:** patient-reported outcome measure, value-based health care, implementation, clinician, behavior, barrier, facilitator, strategies, professionalization, mixed methods

## Abstract

**Background:**

Despite the increasing use of patient-reported outcome measures (PROMs) for collecting self-reported data among hospital outpatients, clinicians’ use of these data remains suboptimal. Insight into this issue and strategies to enhance the use of PROMs are critical but limited.

**Objective:**

This study aimed to examine clinicians’ use of PROM data for value-based outpatient consultations and identify efforts to enhance their use of PROMs in a Dutch university hospital. First, we aimed to investigate clinicians’ use of outpatients’ PROM data in 2023, focusing on adoption, implementation, and maintenance. Second, we aimed to develop insights into the organizational-level strategies implemented to enhance clinicians’ use of PROM data from 2020 to 2023. This included understanding the underlying rationales for these strategies and identifying strategies that appeared to be missing to address barriers or leverage facilitators. Third, we aimed to explore the key factors driving and constraining clinicians’ use of PROMs in 2023.

**Methods:**

We integrated data from 4 sources: 1-year performance data on clinicians’ use of PROMs (n=70 subdepartments), internal hospital documents from a central support team (n=56), a survey among clinicians (n=47), and interviews with individuals contributing to the organizational-level implementation of PROMs (n=20). The Reach, Effectiveness, Adoption, Implementation, and Maintenance framework was used to analyze clinicians’ adoption, implementation, and maintenance of PROMs. Strategies were analyzed using the Expert Recommendations for Implementing Change taxonomy, and results were structured around the constructs of capability, opportunity, and motivation.

**Results:**

On average, around 2023, clinicians accessed PROM data for approximately 3 of 20 (14%) patients during their outpatient consultation, despite numerous strategies to improve this practice. We identified issues in adoption, implementation, and maintenance. The hospital’s strategies, shaped organically and pragmatically, were related to 27 (37%) out of 73 Expert Recommendations for Implementing Change strategies. These strategies focused on enhancing clinicians’ capability, opportunity, and motivation. We found shortcomings in the quality of execution and completeness of strategies in relation to addressing all barriers and leveraging facilitators. We identified variations in the factors influencing the use of PROMs among frequent PROM users, occasional users, and nonusers. Challenges to effective facilitation were apparent, with certain desired strategies being unfeasible or impeded.

**Conclusions:**

Enhancing clinicians’ use of PROMs has remained challenging despite various strategies aimed at improving their capability, opportunity, and motivation. The use of PROMs may require more substantial changes than initially expected, necessitating a shift in clinicians’ professional attitudes and practices. Hospitals can facilitate rather than manage clinicians’ genuine use of PROMs. They must prioritize efforts to engage clinicians with PROMs for value-based outpatient care. Specific attention to their professionalization may be warranted. Tailored strategies, designed to address within-group differences in clinicians’ needs and motivation, hold promise for future efforts.

## Introduction

### Background

Despite the increasing volume of patient-reported outcome measures (PROMs) data collected in hospitals [1], clinicians’ use of these data remains suboptimal [2,3]. PROMs are tools designed to capture patients’ own perceptions of their health, functioning, and quality of life [4,5], often through electronic surveys [1,3]. In outpatient care, clinicians can use a patient’s PROM data to screen and monitor important outcomes and involve patients in medical decisions [6]. In this way, PROMs facilitate value-based health care (VBHC) [7,8], aiming to optimally allocate resources to achieve outcomes that matter to patients.

Achieving and maintaining consistent use of PROM data by clinicians presents difficulties, requiring them to adapt their data use behavior and acquire new skills [3,9,10]. Therefore, to realize the full benefits of PROMs, it is imperative to understand the factors influencing clinicians’ use of PROMs and develop strategies that facilitate and motivate their use in outpatient care [8,10,11].

### Previous Work

Clinicians’ use of PROM data has been proposed as an important metric to evaluate implementation success of PROMs [12]; however, it has received limited attention thus far. The underuse of PROM data is concerning because the effectiveness of PROMs relies on clinicians acting upon the received data [13-16]. Moreover, clinicians’ explicit use is essential for encouraging continued patient participation in future PROMs [17,18].

While comprehensive overviews of barriers to and facilitators of PROMs implementation exist [12,19-21], these often lack a specific focus on clinicians’ perspectives and behaviors [9] and leave gaps in understanding how to respond to the identified factors. While some studies propose hypothetical strategies on the basis of identified barriers [22,23], insights from real-world experiences are limited. A few exceptions provide insights into clinicians’ experiences with PROMs [10,24-27], their self-reported use [24], and implemented strategies [12,20,28]. Thus, there remains a critical need to learn from both successful and less successful implementation experiences aimed at enhancing clinicians’ use of PROMs [10,25,29,30].

### Case

This study draws attention to clinicians’ use of PROMs in a Dutch university hospital, Erasmus Medical Center (Erasmus MC), and the organizational-level strategies initiated to enhance this practice. Adopting a stepwise approach, the first specialty departments began collecting electronic PROMs from their outpatients around 2017, aimed at fostering VBHC [31-33]. New subdepartments continued to join this initiative, continuing into 2024. A central support team (CST) facilitated and coordinated this change, comprising around 6 full-time equivalent and an integrated IT team.

At Erasmus MC, patients complete PROMs before some of their outpatient consultations. To prevent survey overload, a maximum frequency for PROMs completion is set in agreement with subdepartments. This means that during outpatient consultations, clinicians will have a subset of patients for whom new PROM data are available. Clinicians are expected to review these data and discuss them with the outpatient to inform care decisions and actions. This process is facilitated by a consultation room dashboard that visualizes outcomes over time and compares them to reference values. This dashboard shows summary scores as trend lines and allows clinicians to access patient responses to specific items. Clinicians can access this dashboard, which is fully integrated into the patient’s electronic health record (EHR) since 2020, both during preparation for consultations and during the consultations themselves. For the first time, patients will receive feedback on their completed PROMs during their consultation. Individually, they can only access item-level responses without visualizations that aid in interpretation. In future, a separate patient dashboard is planned to offer these additional features.

**Figure 1:**
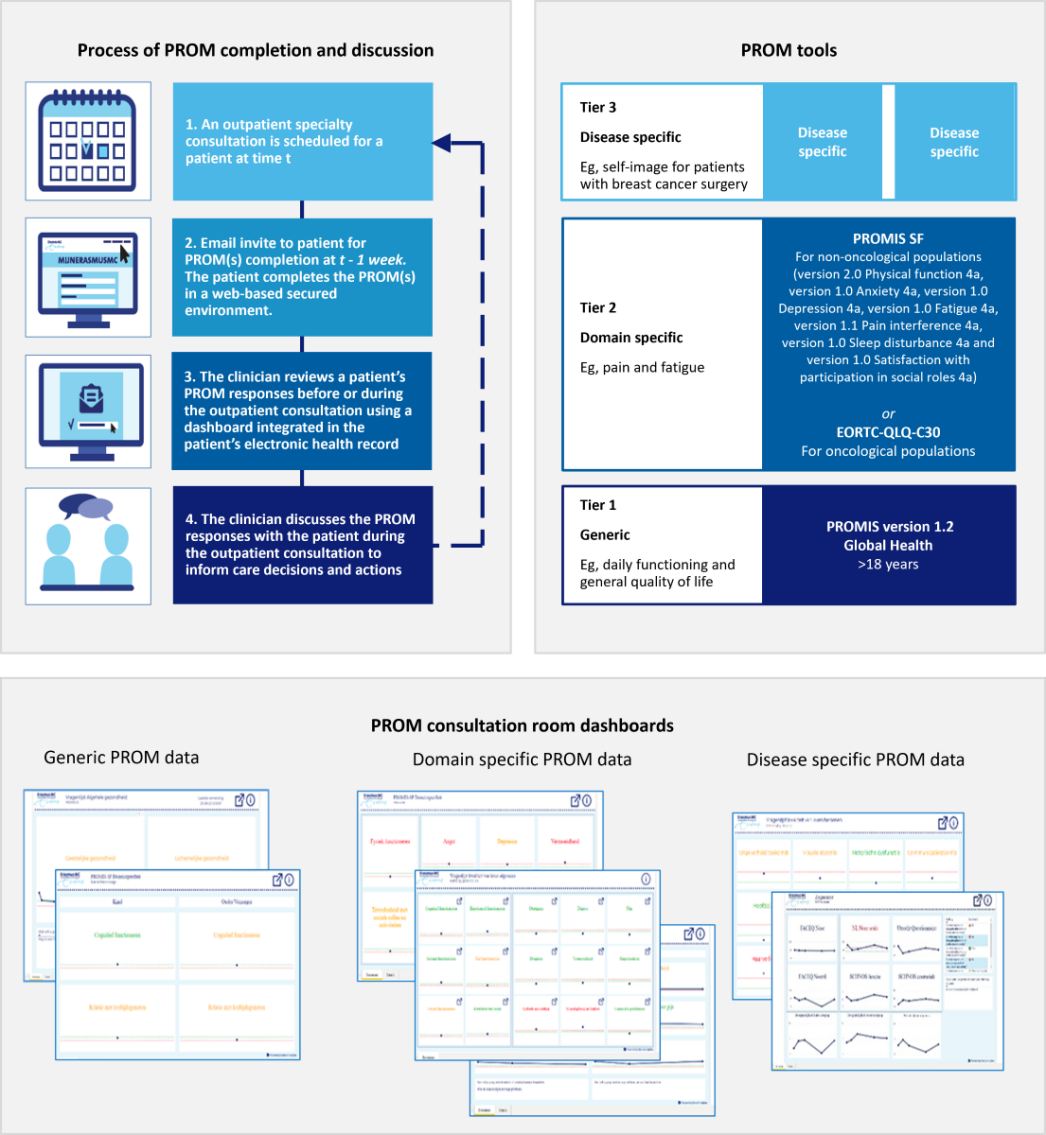
The process of patient-reported outcome measures (PROMs) completion and discussion (top-left), the 3 tiers of PROMs tools used (top-right), and an impression of the dashboard designs (bottom). For the generic tier, the hospital used the Patient-Reported Outcome Measure Information System (PROMIS; version 1.2; Global Health). Within the domain-specific tier, PROMIS short forms (SFs) were used among the nononcological population, including version 2.0, Physical function 4a; version 1.0, Anxiety 4a; version 1.0, Depression 4a; version 1.0, Fatigue 4a; version 1.1, Pain interference 4a; version 1.0, Sleep disturbance 4a; and version 1.0, Satisfaction with participation in social roles 4a. The European Organisation for Research and Treatment of Cancer Core Quality of Life Questionnaire (EORTC QLQ-C30 CAT) was used for the oncological population. Subdepartments could select additional disease-specific PROMs.

Figure 1 [34-36] outlines the process of using PROMs, the specific tools used, and it provides a visual representation of the dashboard. In addition to the PROMs items, the patient survey includes an open-ended question designed to capture topics that the patients wish to discuss during their consultation. Multimedia Appendix 1 provides further information on dashboarding.

By the end of 2023, PROMs were delivered to nearly one-quarter of the outpatient population from 70 subdepartments. However, clinicians’ use of PROM data remained limited. This is despite numerous strategies organically and pragmatically applied by the CST to facilitate and encourage use, including those previously reported as supportive [12,20,28]. This discrepancy highlights the need for further investigation and makes Erasmus MC an intriguing case for investigation.

### Objectives

The objectives of this study are 3-fold. First, we aimed to outline the issues with clinicians’ underuse of outpatients’ PROM data in Erasmus MC around 2023, focusing on adoption, implementation, and maintenance. Second, we aimed to develop knowledge on the organizational-level strategies implemented to enhance clinicians’ use of PROM data from 2020 to 2023. This includes understanding the underlying rationales for these strategies and identifying any strategies that appear missing to address barriers or leverage facilitators. Third, we aimed to investigate the key factors driving and constraining clinicians’ use of PROMs in 2023.

This approach helps understand how implementation unfolds in a real-life context using standardized frameworks to ensure that findings can be compared with other cases and replicated in different settings [37]. In addition, the findings may inform future strategies aimed at facilitating and promoting clinicians’ use of PROMs.

## Methods

### Ethical Considerations

The study was conducted in accordance with the Declaration of Helsinki and exempted from formal approval under the Dutch Medical Research Involving Human Subjects Act by the Medical Research Ethics Committee of the Erasmus School of Health Policy & Management (ETH2223-0225). All participants consented to participation and the publication of quotes. Survey data were collected in a way that ensured participants remained anonymous, and interview data were pseudonymized. All data were stored in a secured space. Participants did not receive compensation.

### Data Sources

#### Overview

This study used 4 sources of data: implementation outcome indicators, a survey, hospital documents, and interviews. Data collection was part of a broader study on the implementation of VBHC [33]. Implementation outcome indicators were used specifically for objective 1, whereas the other data sources were used to address objectives 2 and 3.

#### Implementation Outcome Indicators

The hospital monitored the degree to which clinicians accessed the PROM consultation room dashboard. We extracted the percentage of completed PROMs accessed by clinicians from the hospital’s monitoring system, covering the 12-month period from February 2023 to January 2024. We used the aggregated data that eventually included 70 subdepartments. Investigating subdepartmental differences in the use of PROMs was outside the scope of this study. We analyzed both the average percentage of completed PROMs accessed by clinicians during the week of the patient’s consultation and on the day of the consultation. While the former indicates the use of PROMs to prepare for consultations, the latter serves as a proxy for discussion with the patient.

#### Hospital Documents

Internal hospital documents were accessed through the digital workspace of the CST, to which author VvE was granted access. This workspace was established around 2020. A systematic search was conducted using the following keywords: compliance, evaluation, barrier, facilitator, challenge, plan, intervention, and strategy. This yielded 56 files. These were then screened for data pertaining to factors facilitating or impeding clinicians’ use of PROMs or strategies aimed at enhancing their use, followed by data extraction. If files referred to other files, these were also considered for inclusion. Subsequently, the list of extracted strategies was cross-validated with author MDH-A, a member of the CST, to distinguish between planned and executed strategies.

#### Survey

In January 2023, a collective survey, named EMC23, was digitally distributed to all 194 clinicians across the 35 subdepartments that collected PROM data from outpatients in 2022. One clinician involved in the survey design was excluded from participation. The participants remained anonymous and untraceable. The survey comprised 26 items (Multimedia Appendix 2), developed based on preliminary insight from document analysis. The items delved into clinicians’ self-reported use of PROMs, prominent factors influencing this, and their overall satisfaction with the implementation process and outcomes achieved. In addition, the respondents were asked to evaluate a subset of implemented strategies and had the opportunity to provide comments. Two reminders were sent. As shown in Table 1, 47 responses were included in the analysis.

**Table 1:** Data sources, sample sizes, and participant characteristics.

Data source, theme, and description			Values
**Survey in January 2023 (N=194)**			
	**Respondents, n (%)**		
		Total	57 (29.4)
		Excluded Demographic questions answered (n=5) Not providing patient care (n=2) PROMs^a^ not yet available (n=2) No familiarity with PROMs (n=1)	10 (5.2)
		Complete	42 (21.6)
	**Sex of included respondents (n=47), n (%)**		
		Female	35 (74.5)
		Male	12 (25.5)
	**Age (y), mean (SD; range)**		46 (8.2; 31-64)
	**Function of included respondents (n=47), n (%)**		
		Medical specialist	30 (63.8)
		Nurse	12 (25.5)
		Other (eg, psychologist or resident in training)	5 (10.6)
**Interviews conducted between February 2023 and April 2023 (n=20), n (%)**			
	**Participants**		
		Member of executive board	1 (5)
		Director of quality and patient safety	1 (5)
		Head of VBHC^b^ implementation	2 (10)
		Member of steering committee	2 (10)
		Lead of VBHC program team	3 (15)
		Member of VBHC program team	9 (45)
		External consultant	1 (5)
		Clinician in VBHC	1 (5)
	**Sex**		
		Female	14 (70)
		Male	6 (30)

^a^PROM: patient-reported outcome measure.

^b^VBHC: value-based health care.

#### Interviews

For a process evaluation on the implementation of VBHC [33], author VvE conducted 20 semistructured interviews with individuals involved in the central implementation effort during the past decade (Table 1). Two individuals refused participation for personal reasons. Participants were identified through documents and snowball sampling. Interviews were recorded and transcribed verbatim. Given the hospital’s focus on implementing PROMs in their move to VBHC, interviewees provided valuable insights into the hospital’s efforts and experiences in enhancing clinicians’ use of PROMs. The portion of the interview guide that elicited relevant comments for this study is presented in Multimedia Appendix 3. We reanalyzed these transcripts, extracting information on barriers and facilitators for clinicians to use PROMs, strategies and their rationales, and areas for improvement. These data complemented the other sources mentioned in the Data Sources section, enriching the findings of this study.

### Theoretical Lenses and Data Analysis

#### Overview

**Figure 2:**
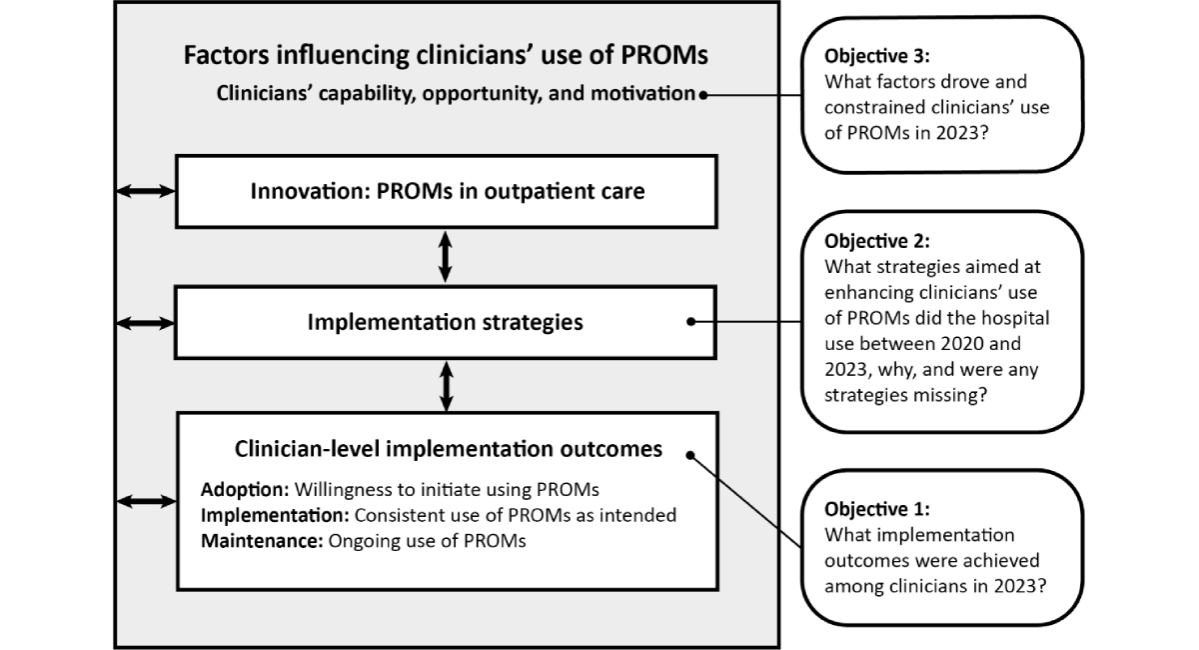
Study objectives. PROM: patient-reported outcome measure.

Given the intricate nature of the implementation of PROMs, adopting a multifaceted research approach is imperative [12], typically including contextual factors, the innovation (PROMs), strategies to support effective implementation of the innovation, and implementation outcomes interact, as illustrated in Figure 2. These elements align with the objectives of this study. Therefore, our methods were plural, incorporating the Reach, Effectiveness, Adoption, Implementation, and Maintenance (RE-AIM) framework [38]; the Expert Recommendations for Implementing Change (ERIC) taxonomy [39] and associated 9 clusters [40]; and the COM-B constructs [41]. We reviewed multiple strategy taxonomies and evaluation frameworks as summarized by Nilsen [42], selecting the aforementioned frameworks due to their alignment with this study’s objectives and data, widespread use, compatibility with the health care setting, and clarity in presenting results. These frameworks were used for post hoc, deductive data analysis; these frameworks are explained in subsequent sections.

#### Objective 1: Implementation Outcomes Among Clinicians in 2023

The RE-AIM framework [38] guides the planning and evaluation of programs by examining 5 outcomes: *reach*, *effectiveness*, *adoption*, *implementation*, and *maintenance*. This study focused on the latter 3 outcomes at the individual level rather than at the department or hospital level. While the collection of PROMs among outpatients was a departmental choice, individual clinicians subsequently needed to adopt, implement, and maintain using PROMs. In this study, *adoption* denoted individual clinicians’ willingness to initiate the use of PROM data from their patients. *Implementation* denoted the consistency of clinicians’ use of PROMs as intended, that is, acknowledging outcomes and discussing results with the patient during their consultation to inform decisions. *Maintenance* referred to the clinicians’ ongoing use of PROMs. *Reach* and *effectiveness* have not been directly studied in this research but were indirectly addressed, as they are contingent upon the extent to which clinicians adopt, implement, and maintain PROM use [8,11].

We examined the outcomes *adoption*, *implementation*, and *maintenance* through *survey* data, reporting item-level frequencies for two key questions: (1) How frequently do you examine patients’ responses to PROMs? with the following response options: *I never used PROMs, I stopped looking at PROMs*, *occasionally*, *as often as possible*, and *always*; and (2) To what extent do you discuss these outcomes with the patient during the consultation? (for those who examine PROMs) with the following response options: *never*, *occasionally*, *as often as possible*, and *always*. We supplemented these data with insights from the hospital’s implementation outcomes indicators, for which no further analyses were required.

#### Objective 2: Implemented and Missing Strategies From 2020 to 2023

We retrospectively analyzed Erasmus MC’s implementation efforts. First, we documented all strategies applied by the hospital from 2020 to 2023 based on *document* and *interview* data, focusing on understanding why these strategies were chosen, particularly in response to barriers or facilitators. For each strategy, we aimed to deductively specify the actor, action, target of the action, temporality, dose, and implementation outcome affected and provide a justification (Multimedia Appendix 4) [43].

Besides describing the practical application of strategies at Erasmus MC, this study mapped discrete ERIC strategies [39] to the identified strategies using a coding manual developed by Fridberg et al [44]. ERIC provides a compilation of 73 discrete strategies that one can use to effectively implement an innovation in a health care setting [39], which have been mapped into 9 strategy clusters [40]. These clusters are mentioned in the Results section. The manual excluded the discrete strategy of facilitation due to overlap with other strategies. This study also included 10 strategies proposed as additions to the ERIC taxonomy: provide stakeholders with the possibility to attend educational meetings [44], recruit clinicians with competence in the innovation [44], provide stakeholders with resources [44], act as a role model [44], assess and redesign workflow [45], create web-based learning communities [45], engage community resources [45], plan for outcome evaluation [46], obtain worker feedback about the implementation plan [46], and communication as a strategy category [47].

To deepen our understanding, we included stakeholder reflections on strategies from *documents*, *interviews*, and *survey* data, including comments on their feasibility and perceived utility. We coded these data by mapping them to the respective strategy they pertained to, allowing us to cluster relevant information accordingly. Subsequently, we integrated these qualitative data into the results per strategy. The dataset was too limited to conduct thematic analyses on a per-strategy basis. Furthermore, we assessed whether identified barriers or facilitators in our data were addressed through these strategies. If not, these cases were labeled as *missing strategies*.

We observed that our data aligned effectively with the constructs of capability, opportunity, and motivation, which are components of the COM-B model [41]. Therefore, we structured our results around these constructs, finding them intuitive and straightforward for conveying our findings.

#### Objective 3: Key Factors Influencing Clinicians’ Use of PROMs in 2023

On the basis of document analysis, we identified key barriers and facilitators to clinicians’ use of PROMs. In the survey, clinicians rated the perceived influence of these factors on their PROM use (Multimedia Appendix 2). They also had the opportunity to identify additional factors.

We analyzed item-level scores for the entire survey population by grouping *agree* and *partly agree* into a single category. Likewise, *disagree* and *partly disagree* were grouped into a single category. The categories *neutral* and *no opinion* remained unchanged. Moreover, we conducted subsample analyses to explore differences among different user profiles. Clinicians were allocated to 3 groups depending on their self-reported use of PROMs: *nonusers*, *frequent users*, and *occasional users*. Nonusers included clinicians who reported never attempting to use PROMs or ceasing to use PROMs. Frequent users comprised those who reported using PROMs as often as possible or always. Clinicians who reported occasional use remained unchanged.

## Results

### Objective 1: Implementation Outcomes Among Clinicians in 2023

#### Implementation Outcome Indicators

The hospital monitored that, on average, from February 2023 to January 2024, clinicians accessed PROM data for nearly 3 out of 20 patients (14%; SD 1.6%) on the day of each patient’s consultation. This data served as a proxy for discussing PROMs with the patient. During the week of the patient’s consultation, which included the use of PROM data for triage and consult preparations, this percentage was slightly higher, averaging at 19.5% (SD 2.4%). While variations in the use of PROM data were apparent across subdepartments and individual clinicians, these specifics were outside the scope of this study.

**Figure 3:**
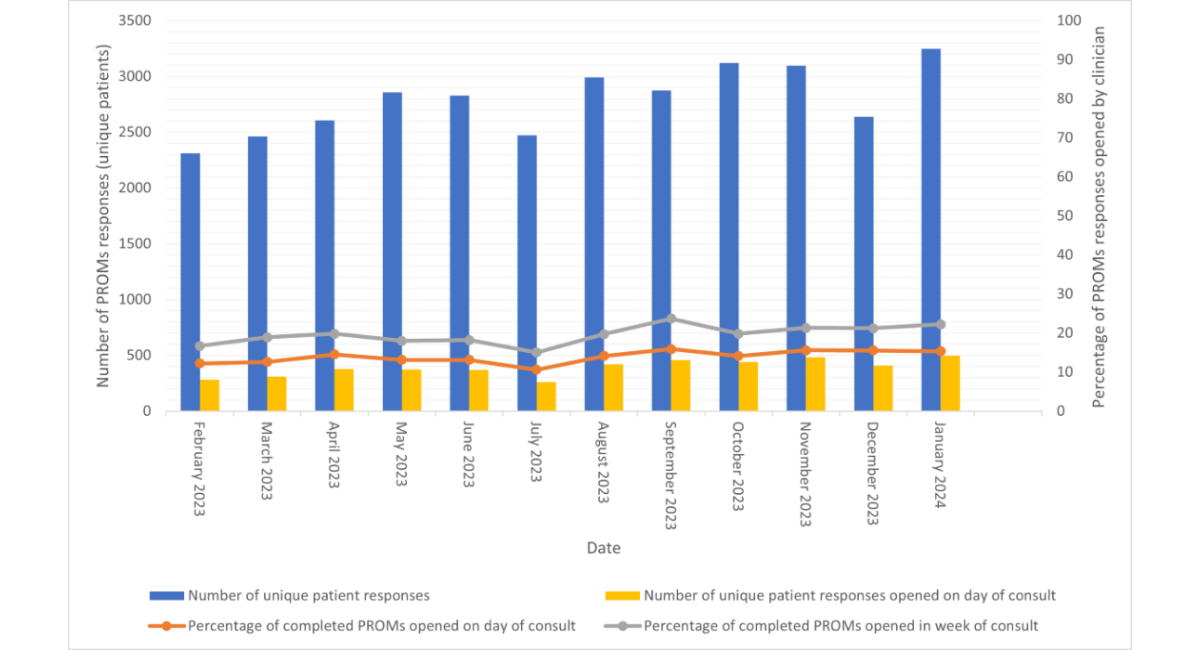
Patient-reported outcome measure (PROM) dashboard use rates among clinicians from February 2023 to January 2024.

Yearly data suggested a slight, albeit minimal, increase in the percentage of completed PROMs of unique patients accessed by clinicians using the dashboard (Figure 3). It is important to note that during 2024, the number of participating subdepartments grew from 38 to 70, implying that around half of the clinicians were in the adoption and implementation stages, while the other half were potentially moving toward the maintenance stage.

#### Survey Data

Clinicians’ self-reported use of PROMs, as derived from our survey in early 2023, revealed issues across all 3 RE-AIM outcomes. Adoption issues were apparent, with around one-tenth (5/47, 11%) of the respondents never attempting to review their patients’ PROMs responses (Table 2). Implementation issues, characterized by inconsistent use of PROMs as intended, became evident, with more than half (27/47, 58%) of the clinicians examining completed PROMs infrequently. Moreover, among those who examined PROMs, less than one-third (10/35, 29%) reported always discussing PROM data with patients. Less than one-fifth of the respondents (6/35, 17%) did not engage in this activity at all. Furthermore, 15% (7/47) ceased examining PROMs, indicating a maintenance issue.

**Table 2:** Clinicians’ self-reported use of patient-reported outcome measures (PROMs).

Items and answers		Respondents, n (%)
**Frequency of examining patients’ responses to PROMs (n=47)**		
	Never attempted	5 (10.6)
	Ceased	7 (14.9)
	Occasionally	17 (36.2)
	As often as possible	10 (21.3)
	Always	8 (17)
**Frequency of discussing PROMs with patients (among those examining PROMs at least occasionally; n=35)**		
	Never	6 (17.1)
	Occasionally	10 (28.6)
	As often as possible	9 (25.7)
	Always	10 (28.6)

### Objective 2: Implemented and Missing Strategies From 2020 to 2023

#### Overview

Table 3 summarizes the strategies that the hospital implemented alongside their corresponding ERIC strategies, structured according to the COM-B constructs of capability, opportunity, and motivation. The hospital used 27 (37%) out of 73 discrete ERIC strategies to enhance clinicians’ use of PROMs alongside 5 proposed additions to the ERIC taxonomy. These are elaborated upon in the subsequent section and specified in Multimedia Appendix 4.

Strategies were drawn from 8 (89%) out of 9 strategy clusters, including using evaluative and iterative strategies, providing interactive assistance, adapting and tailoring to the context, developing stakeholder interrelationships, training and educating stakeholders, supporting clinicians, engaging consumers, and changing infrastructure. No strategies from the cluster financial strategies were used. Qualitatively, it appears that clinicians who used PROMs more extensively rated the supportiveness of the initiated strategies higher than those who used PROMs less. A few strategies were found to be missing, leaving barriers unaddressed or facilitators not leveraged.

Tracking and generalizing strategy specifications, such as temporality and dose, for all strategies was challenging due to the organic and pragmatic application of strategies tailored to specific subdepartment needs and the lack of a comprehensive documented program theory. Having been developed during preceding pilot phases and, most of the strategies were available from the onset of the hospital-wide implementation program in 2020 and refined in the course of time.

**Table 3:** Strategies applied by the hospital, structured around the COM-B constructs of capability, opportunity, and motivation and corresponding strategies from the Expert Recommendations for Implementing Change (ERIC) taxonomy.

COM-B construct targeted by the strategy and strategies by the CST^a^, except otherwise noted			ERIC strategies (except when referenced and noted with N^b^)
**Capability**			
	Conducted a kick-off session and shared a self-developed written and video manual	15^b^. Conduct educational meetings 29. Develop educational materials 31. Distribute educational materials	
	Developed and distributed a pocket guide on discussing PROMs^c^ with patients	29. Develop educational materials 31. Distribute educational materials	
	Developed and conducted training and highlighted external training opportunities	29. Develop educational materials 31. Distribute educational materials 52. Promote network weaving	
	Made training flexible (e-trainings) and sometimes accredited	N1. Provide stakeholders with the possibility to attend educational meetings [44] 29. Develop educational materials 69. Create or change credentialing and/or licensure standards	
	Was accessible for questions and tailored support	43. Make training dynamic 63. Tailor strategies 8. Centralize technical assistance	
	Provided coaching on the job	16. Conduct educational outreach visits 71. Use train-the-trainer strategies	
	Organized peer-to-peer discussion events	20. Create a learning collaborative	
**Opportunity**			
	Created hospital-wide awareness about PROMs and VBHC^d^, aiming to extend reach to extramural parties	N2. Communication [47]	
	Visualized PROMs in a dashboard and integrated this dashboard into the EHR^e^	12. Change record systems	
	Facilitated patients to take the initiative to discuss PROMs	50. Prepare patients to be active participants	
	Facilitated quick actions on PROMs outcomes and streamlined other care processes	12. Change record systems N3. Assess and redesign workflow [45]	
	Implemented a reminder for PROMs in the EHR	12. Change record systems 58. Remind clinicians	
**Motivation**			
	The executive board verbally expressed commitment to achieving VBHC and included it as a hospital aim.	40. Involve executive boards 44. Mandate change N2. Communication [47]	
	The executive board monitored departments’ implementation status and complimented the departments excelling in VBHC.	56. Purposely re-examine the implementation N2. Communication [47]	
	Clinicians participated in the steering committee.	64. Use advisory boards and workgroups	
	The CST communicated about VBHC to achieve and sustain clinicians’ interest.	N2. Communication [47] 41. Involve patients and consumers and family members 7. Capture and share local knowledge 35. Identify and prepare champions 36 Identify early adopters	
	Provided tailored education and held discussions with each subdepartment on PROMs and VBHC	15. Conduct educational meetings	
	Enabled subdepartments to try PROMs with a subset of patients	14. Conduct cyclical small tests of change	
	Aimed to demonstrate the impact of PROMs	N4. Plan for outcome evaluation [46] 24. Develop academic partnerships	
	Gave tailored feedback on subdepartments and clinicians’ use of PROMs	56. Purposely reexamine the implementation 27. Develop and organize quality monitoring systems 48. Organize clinician implementation team meetings	
	Enabled clinicians to adapt PROMs and their uses	51. Promote adaptability	
	Extended the use cases of PROMs	51. Promote adaptability	
	Adapted patient information to alleviate clinicians' concerns about accountability	N2. Communication [47]	
**Generic**			
	Conducted formal and informal evaluations and used the obtained insights to adjust the implementation plan	N5. Obtain worker feedback about the implementation plan [46]	

^a^CST: central support team.

^b^Numbered strategies as per Waltz et al [40].

^c^PROM: patient-reported outcome measure.

^d^VBHC: value-based health care.

^e^EHR: electronic health record.

#### Capability-Associated Strategies

To enhance clinicians’ knowledge and skills in using PROMs, the CST organized kick-off presentations at each department when PROMs were installed. Clinicians received written instructions on using the PROMs dashboard and interpreting outcomes via email, which were later supplemented with video instructions. In 2022, a pocket guide, with exemplary sentences to discuss PROMs was cocreated with clinicians (internal document dated July 14, 2022). Training possibilities, both internal and external, were highlighted. Internal training was often conducted in collaboration with the person-centered care group, focusing on using PROMs in shared decision-making and among patients with limited literacy. The latter aimed to address limitations from clinicians believing that “PROMs cannot be discussed with the patient in question” (internal document, July 14, 2022). Flexible learning resources, such as e-trainings, were developed in response to time constraints:

Attending a conference for half a day can already be challenging, let alone undertaking a lengthy training.

Interviewee 19

In addition, efforts were made to align trainings with clinicians’ accreditation requirements.

However, interviewee 19 noted that training had not been an essential component of the program since 2020; interviewee 10 emphasized a tailored approach that addressed the specific needs of individual subdepartments. The CST increasingly reached out to subdepartments with low use of PROMs. They addressed barriers in perceived capability. One such barrier is clinicians feeling unable to influence certain PROMs outcomes that are affected by external factors, such as divorces impacting the quality of life. This perception led some clinicians to disregard these data (internal document, July 14, 2022). In response, the CST emphasized the importance of empathic listening and referring patients with critical PROMs outcomes to other physicians. Furthermore, since 2022, they provided on-the-job coaching, which proved valuable:

You must take them [a substantial portion of clinicians] by the hand for a moment. Where do you click?

Interviewee 19

They also organized a few peer-to-peer discussion events each year, which interviewee 20 recognized as more impactful than traditional theoretical instruction. However, attendance issues arose with clinicians not always able to participate in these sessions. While survey results indicated that around 60% (26/43) of respondents were dissatisfied with the training or coaching received, skill issues were not a prominent factor impeding their use of PROMs (Table 4).

**Table 4:** Prominent reasons for clinicians to use patient-reported outcome measures (PROMs), both in total and as per 3 user profiles.

Item, post hoc categories, and answer options				All respondents who used PROMs at least once (n=42)		User profiles					
						Nonusers (n=7)^a^		Occasional users (n=17)		Frequent users (n=18)	
**Most prominent factors or reasons for why I used PROMs, n (%)**											
	**Motivation**										
		Benefits for the patient	16 (38)		0 (0)		5 (29)		11 (61)		
		Benefits for myself	15 (36)		0 (0)		5 (29)		10 (56)		
	**Opportunity**										
		It is expected from me.	19 (45)		3 (43)		9 (53)		7 (39)		
		Patient requests	5 (12)		2 (29)		3 (18)		0 (0)		
**Calculation**											
	Number of factors selected, mean (SD)			1.3 (0.9)		0.7 (0.5)		1.3 (1.0)		1.6 (0.9)	

^a^There are lower sample population values compared to the total population value because this question was posed to ceased users only (n=7), excluding those who never attempted PROMs (n=5).

#### Opportunity-Associated Strategies

Another set of strategies targeted clinicians’ social and physical environments to create opportunities for using PROMs. Regarding clinicians’ social context, the CST aimed to create legitimacy and enable clinicians to discuss PROM data during multidisciplinary consultations, even with colleagues who had not yet initiated PROMs. They focused on raising hospital-wide awareness about PROMs and VBHC through mass communication via email, intranet, and marketing in public spaces. One clinician described a challenge in this endeavor:

Emails get buried under more important ones, and newsletters go unread. However, too little communication also doesn’t seem to work (you never do it right).

Internal document, August 31, 2020; clinician

Interviewee 14 also noted difficulties arising from the organization’s size, with hard-to-reach *islands*.

Anticipated future strategies include providing information about PROMs to general practitioners and external providers involved in patient care. Clinicians requested this strategy to facilitate communication with external health care professionals, such as making them aware of possible referrals on the basis of PROMs scores. However, this strategy was deemed feasible once PROMs were used among all patients, as partial implementation would require excessive manual effort (internal document, November 23, 2020; interviewee 19). Furthermore, a dashboard enabling patients to review their own outcomes will be developed to increase patient initiative in discussing PROMs:

If patients can see their outcomes, they’ll probably ask more questions about it. So, it will get used more by clinicals, both intentionally and unintentionally.

Interviewee 2

Currently, to encourage patient initiative, patients are provided with resources such as an animated video explaining how to discuss PROMs during consultations.

Regarding the physical environment, several clinicians experienced or assumed PROM use in outpatient care as incompatible with their workload:

It takes far too much time, and we don’t get any extra.

Survey respondent 8

To mitigate this issue, the hospital sought to streamline the care process and simplify PROM use, as extending available consultation time was not feasible. Interviewee 10 explained as follows:

Small adjustments can make a real difference for a doctor who may not necessarily be receptive to changing their practice.

As per interviewee 15, integration of the PROMs dashboard into the EHR was considered a major improvement. Two other features reduced the registration burden: quick copying of PROMs scores into patient notes and a referral template for easily referring patients needing further attention on the basis of PROM outcomes (internal document, May 11, 2023). However, 79% (33/42) of the survey respondents had no opinion on the usefulness of the referral letter, possibly indicating unawareness of this feature. In addition, the CST implemented a reminder by prominently displaying the PROMs completion status on the patients’ EHR front page. This also eased the workflow by eliminating the need to open the PROMs dashboard to verify completion. More than one-third (15/42, 36%) of the survey respondents perceived this feature as helpful.

Moreover, to save time, PROM data were used in triage and the developed PROMs-IT infrastructure was used to have patients report their medication and lifestyle. This extension reduced the necessity for discussions on these topics during consultations (internal document, May 11, 2023). Despite these efforts, perceived time constraints remained the most frequently cited barrier among clinicians as of early 2023 (Table 4). Loading time delays of the PROMs dashboard exacerbated these constraints, resulting in decreased commitment and temporary cessation of PROM use. These delays were resolved, and a dedicated campaign was organized to encourage the reuptake of PROMs.

#### Motivation-Associated Strategies

The use of PROMs remained nonobligatory, focusing on genuine engagement rather than mere compliance. The inclusion of clinicians in the steering committee aimed to create “visible standard-bearers from within the healthcare sector” (internal document, May 20, 2019) to enhance buy-in. The CST frequently communicated about PROMs and VBHC to achieve and sustain interest. They shared stories highlighting the benefits of PROMs from both patients and clinicians via a monthly newsletter and intranet. One of the ideas was that “if it’s beneficial for the patient, it usually has added value for the clinician as well” (internal document, July 14, 2022). Stories of championing clinicians were noted as a key facilitator in documents and interviews (internal document, August 31, 2020; interviewee 3). However, approximately half (21/43, 49%) of the survey respondents did not feel that the stories of other health care professionals positively influenced their attitude toward working with PROMs, with only 16% (7/43)—primarily consistent PROM users—reporting that they did. In communication, the CST also emphasized the importance of clinicians’ discussion of PROMs on patient adherence to complete subsequent PROMs, supported by local evidence (internal document, June 7, 2023). Sometimes, the CST chose not to overtly reference VBHC, focusing instead on visible outcomes for clinicians, such as PROMs enhancing the patient-clinician conversation. Interviewee 3 explained as follows:

Sometimes your sales pitches need to be much flatter than what’s underneath it all.

This adjusted framing aimed to address resistance among clinicians who viewed VBHC sceptically, perceiving it, for example, as a “dull, container concept” (interviewee 6) and “a hype” (survey respondent 11). As another reason, terminology shifts in the national policy created confusion and was overwhelming (interviewee 3). Nonetheless, some clinicians perceived the hospital’s communication efforts negatively. Survey respondent 60 mentioned, “extremely selective advertising,” and survey respondent 11 noted that “a hallelujah story is being told that overlooks the differences in patient populations.”

Concurrently, formal pressures increased: the executive board verbally committed to achieving VBHC, supported by video messages and integration into hospital aims. Externally, government programs supported PROMs and VBHC [48,49], and external audits increasingly evaluated PROM use (internal document, July 14, 2022). These factors directly motivated clinicians and empowered department heads to prioritize PROM use among their employees, according to interviewee 14. Since 2022, the executive board requested subdepartments to report on their VBHC implementation status thrice yearly and complimented high-performing departments (internal document, July 14, 2022). The CST proposed that the executive board explicitly request information on clinicians’ use of PROMs rather than merely their inquiries among outpatients (interviewee 19). However, this proposal has not been implemented thus far, and the reasons for this remain unknown to the CST. Survey respondent 11 expressed concerns about such an approach:

My biggest fear is that it [PROMs] becomes an outcome, that the departments that use it are deemed “better” than those that do not.

This fear may have stemmed from clinicians’ belief that PROMs do not suit all patient groups or consultations equally (internal document, July 14, 2022).

To facilitate adoption, subdepartments were enabled to try PROMs with a subset of patients. Interviewee 19 highlights the importance of clinicians developing direct experience with PROMs:

[T]he true impact of PROM data becomes evident when you experience it.

However, implementing PROMs among a subset of patients had an unintended consequence:

If we decide, “We’ll do it for X and Y but not for Z”... well, then you forget about it.

Interviewee 7

Forgetting to use PROMs was the second-most cited barrier by clinicians (Table 4). To stimulate sufficient PROM survey volume, several strategies aimed to enhance patient response rates to PROMs. Furthermore, the CST aimed to evidence the benefits of PROMs and their impact on professionals’ work experience, including time investment. Interviewee 6 stated the following:

The key question is, do you have proof? That is the ultimate killer question.

However, these efforts were hindered by limitations in defining measurable outcome measures, data availability, and difficulties in consolidating local evidence and making it convincing to other disciplines. Moreover, the hospital conducted both formal and informal evaluations of PROMs among clinicians to gain insights into their experiences, motivations, and needs, subsequently adapting implementation plans on the basis of these findings. This included prioritizing disease-specific PROMs, extending training opportunities, and expanding PROM data use to areas beyond outpatient care, such as triage, research, and care pathway improvements, tapping into different clinician motivations (interviewee 17). In addition, the CST alleviated clinicians’ concerns about accountability by adapting patient communication to clarify patients’ responsibility for timely contact in critical situations via traditional means.

To achieve and maintain effective implementation, the CST provided feedback on PROM use to subdepartments. The frequency and format of feedback was tailored to departmental preferences, with some favoring nonanonymous feedback, such as *“*[clinician’s name] has accessed this many PROMs” (interviewee 19), for a competitive aspect, while others preferred anonymity. However, nearly half of the survey respondents (20/43, 47%) experienced infrequent feedback. Furthermore, local departments were increasingly empowered to adapt PROMs and their application to their context, addressing limitations of uniform solutions on buy-in, perceived ownership, and local fit (interviewee 18). Subdepartments could incorporate disease-specific items that were deemed critical:

[a generic PROMs] is excellent for (big) data collection but lacks clinical value; it’s too general.

Internal document, August 31, 2020

Moreover, they could determine patient eligibility criteria, adjust inquiry frequency, and choose from multiple dashboard layouts (internal document, May 11, 2023). Individual clinicians were also enabled to manually exclude patients from PROMs if needed, aiming to mitigate challenges from upset patients:

The feeling of incompetence [to complete a PROM] among the less literate population takes a huge toll on their outpatient visits.

Survey respondent 60

Even the son of one of my patients complains about it.

Survey respondent 7

Moreover, it aimed to overcome a specific drawback noted by survey respondent 4:

[Patient] inclusion in studies is less due to PROMs, so that irritates me.

Overall, interviewee 20 perceived the following:

Once you give a team the feeling of autonomy or autonomy itself, then you already win 80% of the battle to get them to use it.

#### Missing Strategies

Unaddressed barriers also appeared in relation to clinicians’ capability, opportunity, and motivation. Various interviewees highlighted shortcomings in contemporary education, noting a lack of emphasis on developing the professional attitudes necessary for effectively using PROMs in VBHC. This includes insufficient focus on understanding the impact of disease on the individual, their social environment, and work (interviewee 20), as well as limitations in shared decision-making, inclusive care, and data-driven improvement work (interviewee 19). Interviewee 14 pointed out that VBHC requires clinicians to take shared responsibility for patient outcomes rather than dismissing certain issues as outside their scope, observing that this attitude is “only really embraced by a few people.” Interviewee 18 emphasized another limitation in education, stating the following:

It has become checkbox medicine.... [Residents are] afraid to color outside the lines of those protocols, guidelines, and contractual agreements.

In addition, there seem to be limitations in clinicians’ capacity for change and medical leadership (internal document, July 13, 2020). Interviewee 11 noted that clinicians are typically “quite conservative” and find changing behavior “very difficult.” Interviewee 20 observed that PROMs resonate more with nurses, who focus more on caring and benefit from longer consultation times, while physicians typically focus on curing. The CST found that departments with a dedicated nurse specialist or physician assistant achieved higher use of PROMs compared to those without such staff. Some subdepartments tasked these nurses with discussing patients’ PROM data and flagging any issues for the medical specialist who would see the patient next (internal document, May 11, 2023). However, the CST struggled to leverage this facilitator across the hospital due to budget constraints in local subdepartments to hire nurse specialists or physician assistants and the nonbillability of PROMs discussions by nurses. Overall, the CST believes that patients’ experiences of symptoms, functioning, and quality of life should be a central focus in all care activities rather than being narrowed to nurse consultations alone.

There also appeared limitations in the structural integration of PROMs for VBHC into the onboarding of new staff. Survey respondent 39 noted the following:

I started working here a year ago, there hasn’t been a structured introduction to this topic, I have no experience with it.

Interviewee 19 explained that despite 2 attempts by the program team to include PROMs in onboarding, these efforts faced rejection due to PROMs not yet being uniformly adopted across all departments.

Behavior change was further hindered by limitations in opportunity and motivation. Clinicians’ contemporary feelings of work pressure and well-being were considered to have a negative impact:

People feel exhausted, like stretched rubber bands nearing their breaking point.... They think: “What now again? Will this add further strain to me?”

Interviewee 6

Interviewee 19 noted the challenge of change fatigue alongside “a pervasive aversion to any form of registration and data handling.” A fine line existed between meeting data needs and experiencing an overwhelming availability of data. Interviewee 11 noted a lack of tension and urgency to use PROMs:

There is no fire. There are no patients dying if you don’t use PROMs.

Interviewee 18 noted the challenge from the ongoing volume-based rather than value-based payment, impeding clinicians’ opportunity to use PROMs to make value-based decisions:

We ask people to play a different game of soccer without changing the rules. Resultantly, no one ends up playing differently.

### Objective 3: Key Factors Influencing Clinicians’ Use of PROMs From Early 2023

#### Overview

Despite implementing numerous strategies (objective 2), the hospital continued to face limitations in clinicians’ use of PROMs (objective 1). Therefore, as the third objective, we aimed to broaden our understanding of the key factors influencing clinicians’ use of PROMs in early 2023. We first highlight the primary motivations driving clinicians to use PROMs, followed by the key factors contributing to suboptimal use.

#### Reasons to Use PROMs

Survey respondents, excluding those who never attempted using PROMs, were asked to select the most prominent reasons for using (or having used) PROMs. Response options were identified from document analysis. Respondents had the opportunity to add additional factors, but this option was not used.

Among all survey respondents, 38% (16/42) reported using PROMs primarily because of patient benefits and 36% (15/42) reported using it because of personal benefits (Table 4). Nearly one-third (12/42, 29%) experienced both types of benefits. In addition, 45% (19/42) of respondents used PROMs because they felt it was expected of them, with 31% (13/42) citing this expectation as the sole reason for using PROMs. Only 12% (5/42) used PROMs because of patient requests.

Table 4 also shows responses among 3 user profiles: *nonusers*, *occasional users*, and *frequent users* (refer to the Methods section). Perceived benefits were the primary driver for frequent users, while feeling expected to use PROMs was the most frequently selected reason among occasional users and those who ceased use. Frequent PROM users selected more factors compared to less frequent users, suggesting that they perceived more reasons to use PROMs.

#### Barriers and Satisfaction

Survey respondents were also asked to select the most prominent factors that impeded their use of PROMs, if experienced, in a multiple-choice question. Among all respondents, key issues were time constraints (24/44, 55%) alongside dashboard functioning limitations (24/44, 55%), followed by forgetting (22/44, 50%; Table 5). Limitations in reinforcement and believing to have a limited impact on improving PROMs domains were rarely selected as reasons impeding PROM use.

Disaggregating into the 3 user profiles, we found that dashboard functioning limitations impeded all groups. While all nonusers perceived time constraints, this factor was less frequently selected among the other profiles. Nonusers often cited a misalignment between PROMs and how they preferred to work as a substantial factor. Among occasional users, forgetting to use PROMs was a critical issue, a challenge also faced by current nonusers. The frequent PROM users felt impeded by the low volume of completed PROMs. Overall, those who used PROMs less frequently or not at all selected more factors compared to more frequent users.

Respondents were also asked to grade the outcomes of implementing PROMs in outpatient care and the implementation process; respectively, average scores were 4.9 and 5.4, both out of 10. Clinicians who used PROMs more frequently were more satisfied (Table 5).

**Table 5:** Prominent factors that contribute to suboptimal use of patient-reported outcome measures (PROMs), if experienced, both in total and as per 3 user profiles.

Item, post hoc categories, and answer options			All respondents (n=44^a^)	User profiles		
				Nonusers (n=11^a^)	Occasional users (n=15^a^)	Frequent users (n=18)
**Most prominent factors contributing to suboptimal use of PROMs, if experienced; n (%)**						
	**Capability**					
		Finding it difficult or perceiving not to possess necessary skills	8 (18)	3 (27)	4 (27)	1 (6)
	**Opportunity**					
		Dashboard functioning issues	24 (55)	8 (73)	8 (53)	8 (44)
		Not enough time	24 (55)	11 (100)	8 (53)	5 (28)
		Low volume of completed PROMs	17 (39)	4 (36)	5 (33)	8 (44)
		A colleague discusses PROMs^b^	2 (5)	0 (0)	2 (13)	0 (0)
	**Motivation**					
		Not in my routine, I forget it	22 (50)	8 (73)	10 (67)	4 (22)
		Misalignment with how I prefer to work	12 (27)	6 (55)	5 (33)	1 (6)
		No added benefits for patients or myself	11 (25)	3 (27)	5 (33)	3 (17)
		PROMs do not fit my patient population	7 (16)	3 (27)	2 (13)	2 (11)
		I have limited influence on improving PROM domains	4 (9)	0 (0)	3 (20)	1 (6)
		Too little reinforcement	2 (5)	(18)	0 (0)	0 (0)
**Calculation**						
	Number of factors selected, mean (SD)		3.0 (1.7)	4.4 (1.3)	3.5 (1.6)	1.8 (1.1)
**Average grade from 1 to 10**						
	PROMs implementation process, mean (SD, range)		5.4 (2.4, 1-9)	2.7 (1.7, 1-6)	5.3 (1.8, 3-8)	7.1 (1.6, 4-9)
	PROMs implementation, mean outcomes (SD, range)		4.9 (2.3, 1-9)	2.7 (1.9, 1-6)	4.8 (1.6, 2-8)	6.2 (1.9, 3-9)

^a^There are lower sample population values compared to the total population value due to missing data from incomplete responses.

^b^Some local departments adapted professional roles and workflows (strategy addition [45]) so that physician assistants or nurses were responsible for discussing patients’ PROM data and signaling any issues that require attention to the medical specialist who sees the patient next (internal document, May 11, 2023).

## Discussion

### Overview

Clinicians’ effective use of PROM data is crucial for maximizing the benefits of PROMs and ensuring their ongoing use [13-17]; however, it remains challenging to achieve [3,9,10]. This study aimed to (1) explore how clinicians use individual patients’ PROM data for value-based outpatient specialty care in a Dutch university hospital, (2) identify organizational strategies used to enhance PROM data use, and (3) uncover key motivations and barriers that persist.

Our findings indicate that clinicians’ use of PROM data remains suboptimal despite numerous strategies aimed at enhancing their capability, opportunity, and motivation. We identified shortcomings in the execution quality and comprehensiveness of applied strategies. Factors that influenced PROM use varied among clinicians, and some strategies were hindered or controversial, adding to the complexity of effective change facilitation.

### Clinicians’ Use of PROMs

On average, between early 2023 and 2024, clinicians accessed PROM data for approximately 3 of 20 (14%) patient responders on the day of the patient’s consultation, serving as a proxy for discussing outcomes during the consultation. We found clinicians not trying PROMs (an adoption issue), using PROMs inconsistently (an implementation issue), and stopping the use of PROMs (a maintenance issue), underscoring the inherent challenges in altering clinicians’ practices and the need for multifaceted strategies. The persistent challenges in PROM use rates, both in our study and others [3], contrast with a study reporting higher self-reported use rates [24] and clinicians prioritizing discussions about PROMs in outpatient consultations [17].

### Strategies to Enhance PROM use

The hospital used 27 (37%) out of 73 ERIC strategies [39] to enhance clinicians’ use of PROMs, with the only category not used being financial strategies [40]. Our study also confirmed 5 strategies that scholars proposed as additions to ERIC: communication [47], providing stakeholders with the possibility to attend educational meetings [44], assessing and redesigning workflow [45], obtaining employee’s feedback about the implementation plan [46], and planning for outcome evaluation [46].

Strategies appeared to address clinicians’ capability, opportunity, and motivation to use PROMs, aligning with the crucial constructs in the COM-B model.

Regarding *capability*, the results of this study confirmed that training clinicians in using PROMs for VBHC is crucial [27]. This underscores the need for integration into (interprofessional) education and ongoing training efforts for current staff as well as effective onboarding for new employees. Effective use of PROMs appears to require more substantial and impactful changes than initially anticipated, requiring a shift in clinicians’ roles, professional attitudes, and ingrained practices. These changes include extending accountability and collaboration as well as focusing on care in addition to cure. There appears need for attention to professionalization [50,51], including the development of change capability [52] and nurse or medical leadership [51,53-55]. For training current staff, individualized approaches such as coaching on the job appeared helpful. Moreover, attention should be given to providing clinicians with opportunities for training, considering their busy schedules [44].

Regarding *opportunity*, strategies were directed at creating both social and physical opportunities to use PROMs. The hospital made substantial efforts to simplify and streamline the use of PROMs by adopting strategies identified by other scholars as supportive, such as integrating PROMs into the EHR with graphical interpretation support [10,15,25,56,57], quick copy of PROMs outcomes into patient notes [57], and using PROMs to collect previsit information on medication and lifestyle [56]. Nevertheless, more than half (24/44, 55%) of the clinicians surveyed in early 2023 cited time constraints as a barrier to using PROMs, alongside a temporary issue of long loading times of the PROMs dashboard. Time has been reported as a barrier to PROM use previously [7,24,27]. While PROMs may take more time due to inexperience [31,58], research has also shown that PROMs do not lengthen consultation time [59,60] and some clinicians experience time savings [7,58]. These discrepancies warrant attention. Possibly, emphasis may be needed to manage clinicians’ assumptions and perceptions of time. Moreover, it is critical that PROMs and associated dashboarding function as needed, representing a cornerstone. In addition, the current payment system hinders the use of PROMs, requiring clinicians to focus on outcomes while reimbursing based on volume of health services. Moreover, it restricts the roles nurses can assume regarding PROMs, as their PROMs discussions are not always reimbursable.

Regarding motivation, most clinicians were driven by the perceived benefits of PROMs for both themselves and their patients. Smaller groups were motivated to use PROMs due to role expectations and patient–initiated discussions, making these potential levers that hospitals could tap on. This may be particularly important to address, as prior research suggests that professionals may expect patients to initiate discussions about PROMs, while patients believe this responsibility lies with the professionals [26]. We recommend that hospitals clarify the motivations for implementing PROMs in outpatient care and encourage professionals to explore and be driven by their own motivations. Conversely, we found that a quarter (12/44, 27%) of clinicians, especially those who have now quit using PROMs, did not perceive PROMs to align with how they prefer to work. This highlights a potential area for further research.

Challenges also seem to stem from local circumstances, with staff feeling pressured and lacking the tension and evidence needed to change. By measuring patient and professional outcomes and experiences, such as the quality of conversations, pre-PROMs implementation and post-PROMs implementation [61], hospitals could instill a sense of urgency for change and provide the requested evidence.

The positioning of PROMs as either a voluntary tool or a core element of care impacts the range of strategies that can be used. In the observed case, PROM use remained voluntary to prevent their ceremonial use [62]. However, there was an increase in formal pressures, both internally and externally, including government focus on outcomes and the value of care [48,49]. To address limitations related to clinicians viewing PROMs and VBHC as supplementary rather than integral to their work, hospitals may consider embedding these practices into their core operations. To exemplify, integrating PROMs and VBHC into hospital strategy, communications, education, career development discussions, control cycles, and mainstream IT could facilitate institutionalization [50,63].

### Challenges in Strategy Selection and Implementation

The hospital selected and implemented strategies to enhance the use of PROM use in a pragmatic and organic manner without a comprehensive and detailed documented program theory. They did not evaluate the effectiveness of the strategies used. However, finding linear and singular cause-and-effect linkages may be unfeasible given the complexities of change [64]. Complex change typically includes multiple and interacting factors influencing behavior as well as characteristics of nonlinearity and path dependency [64-66].

Challenges in effective change facilitation included unfeasible strategies due to limitations in manpower and budget as well as constraints inherent in the staged implementation approach. For example, the latter necessitated a simultaneous focus on adoption, implementation, and maintenance. Moreover, certain strategies had unintended consequences. For example, initially implementing PROMs for a subset of patients facilitated adoption but ultimately led to infrequent use. Half (22/44, 50%) of the clinicians cited forgetting to use PROMs because it is not a part of their routine, which echoes prior findings [27] and presents a substantial risk to maintaining PROM use [67]. Scheduling consultations for which PROMs are sent together may be a potential solution worth investigating further.

Furthermore, we identified challenges stemming from the diffusing nature of factors influencing clinicians’ use of PROMs. We identified variations in critical factors among frequent users, occasional users, and those who have never attempted or have quit using PROMs. For instance, nonusers more often cited perceived time constraints as a prominent barrier compared to frequent users and felt that PROMs did not align with their preferred way of working. Consequently, increased understanding and tailoring of strategies on the basis of behavioral determinants appears promising. In addition, we anticipate that the hospital’s approach could benefit from increased focus on achieving higher levels of workforce activation [68]. Above all, a CST can only facilitate and encourage but not manage clinicians’ use of PROMs; each individual clinician should ultimately be engaged and take leadership in using PROMs.

### Future Research

Future research could explore the factors contributing to differences in the adoption, implementation, and maintenance of PROMs across subdepartments. Conducting interviews could help delve deeper into specific findings, particularly in areas such as routines, time perceptions, and professionalization. Investigating the association between clinicians’ characteristics and their use of PROMs would be valuable. Deeper exploration of specific strategies, including their adaptations over time, for example, using Justin Smith’s Longitudinal Implementation Strategy Tracking System [69], could also provide valuable insights.

### Limitations and Strengths

Regarding the scope of the study, we focused on clinicians’ use of PROM data, but their role is broader and likely includes motivating nonresponding patients to complete subsequent PROMs. We focused on reporting whether strategies were used rather than evaluating their execution and effectiveness. Our emphasis on organizational-level strategies led us to overlook decentralized strategies and their potential interactions. Regarding our data sources, the low survey response rate necessitates cautious interpretation. However, survey respondents reported varying frequencies of PROM use and satisfaction, which suggests the inclusion of a diverse range of perspectives. The timing of the survey followed a period of limited dashboard functioning, which likely influenced the results. Interview data were collected as part of a broader research question, so not all respondents systematically provided input on strategies and challenges associated with PROM use. This limitation hindered our ability to quantify the percentage of interviewees supporting specific arguments and potentially limited completeness. In addition, there may be missing documents that were not captured by the screening keywords. The specification of strategies and the application of ERIC coding may be open to interpretation and hence introduce bias. However, we aimed to enhance rigor by using a coding manual.

Despite these limitations, our study is a pioneering effort to investigate the underuse of PROMs and document practice-developed strategies to enhance their use among clinicians. Furthermore, our study uniquely highlights how implementation factors impact clinicians differently in a hospital setting, suggesting the potential benefit of tailored strategies.

### Conclusions

Achieving clinicians’ use of PROMs in value-based outpatient care has remained challenging despite various strategies aimed at enhancing their capability, opportunity, and motivation. Their experienced barriers and motivations were diverse and sometimes difficult to address, adding to the complexity of effective facilitation. PROM use may require more substantial changes than initially expected, necessitating a shift in clinicians’ professional attitudes and practices. Hospitals can facilitate and encourage rather than manage clinicians’ genuine use of PROMs. They must prioritize efforts to engage clinicians with PROMs for value-based outpatient care. Specific attention to their professionalization is warranted. Tailored strategies designed to address differences in clinicians’ needs and motivation hold promise for future efforts and could also facilitate the successful implementation of other eHealth initiatives.

## Appendix

Multimedia Appendix 1 Patient-reported outcome measure dashboarding visualizations.

Multimedia Appendix 2 Survey.

Multimedia Appendix 3 Semistructured interview questions.

Multimedia Appendix 4 Specification of strategies.

## Abbreviations

CST: central support team
EHR: electronic health record
Erasmus MC: Erasmus Medical Center
ERIC: Expert Recommendations for Implementing Change
PROM: patient-reported outcome measure
RE-AIM: Reach, Effectiveness, Adoption, Implementation, and Maintenance
VBHC: value-based health care

## Data Availability

The datasets generated during and analyzed during this study are available from the corresponding author on reasonable request.
