# Supplementary material for: Enhancing Clinicians’ Use of Electronic Patient-Reported Outcome Measures in Outpatient Care: Mixed Methods Study
Source: J Med Internet Res. 2024 Oct 18;26:e60306. doi: 10.2196/60306 (PMC11530726; doi:10.2196/60306)
Supplement: Multimedia Appendix 1 [file jmir_v26i1e60306_app1.docx]

**Appendix 1. PROMs dashboarding visualizations**

PROM scores are calculated per tool according to the guidelines of the individual tools and visualized in a dashboard (see Figure 1). The dashboard shows patients’ PROMs score per domain (over time if longitudinal data is available) and enables clinicians to view item responses.


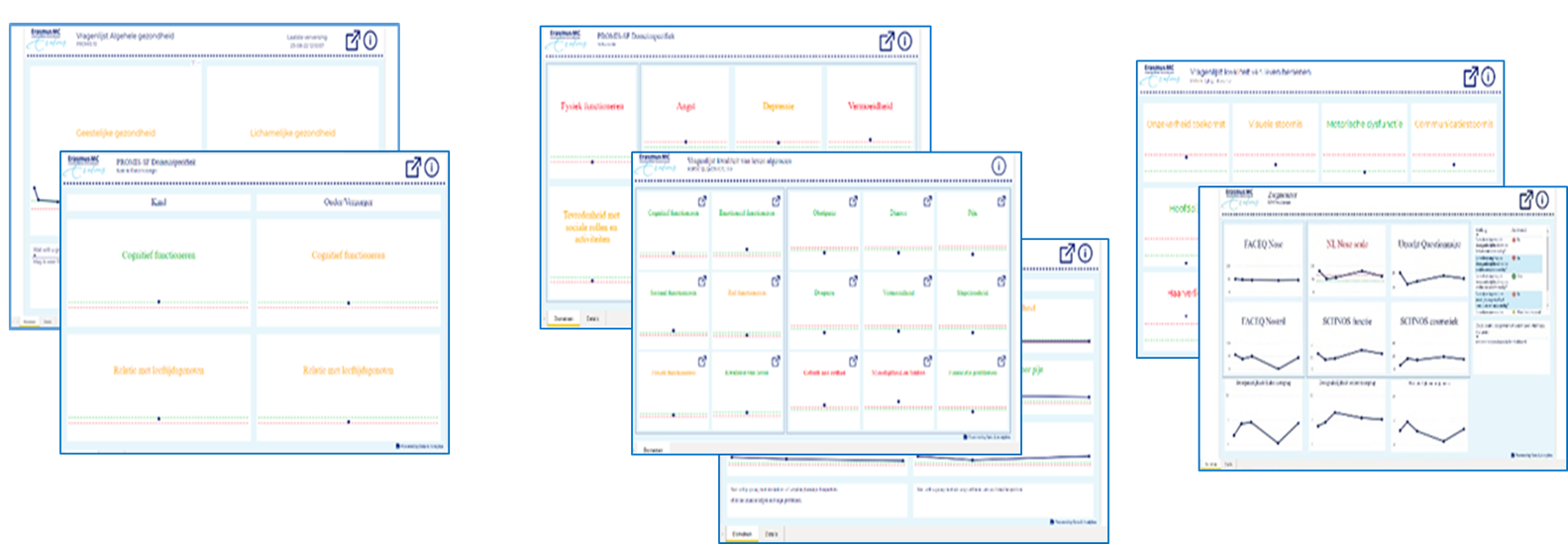


**Figure 1**. PROM dashboards (left: generic PROMs, middle: domain specific PROMS, right: disease-specific PROMs) for clinician and patient to review during the consulting.

The colors green, orange and red are used to provide instant insight in whether domains do or do not warrant attention. For this visualization, one of the following three methods are used:

**Method A.** When Dutch reference values are available:

- If a higher score indicates better patient status:
  - Color red: Lowest possible score up to the mean minus 2 standard deviations of the reference population
  - Color orange: Between "mean minus 2 standard deviations" and "mean minus 1 standard deviation" of the reference population
  - Color green: From "mean minus 1 standard deviation" of the reference population and higher
- If a higher score indicates worse patient status:
  - Color red: Highest possible score up to the mean plus 2 standard deviations of the reference population
  - Color orange: Between "mean plus 2 standard deviations" and "mean plus 1 standard deviation" of the reference population
  - Color green: From "mean plus 1 standard deviation" of the reference population and lower

**Method B.** When no reference values are known (certain disease-specific dashboards), expert values are determined in consultation with the department, often based on the following principles:

- If a higher score indicates better patient status:
  - Color red: 0% – 24% of the total possible score
  - Color orange: Between 25% – 75% of the total possible score
  - Color green: Between 76% – 100% of the total possible score
- If a higher score indicates worse patient status:
  - Color green: 0% – 24% of the total possible score
  - Color orange: Between 25% – 75% of the total possible score
  - Color red: Between 76% – 100% of the total possible score

**Method C**. In disease-specific dashboards, deviations from methods A and B are sometimes made when certain symptoms are always considered alarming. In such cases, the presence of the symptom always scores red. For example, swallowing difficulties in head and neck oncology or coughing up blood in lung oncology will always be shown as red.
