# Supplementary material for: Enhancing Clinicians’ Use of Electronic Patient-Reported Outcome Measures in Outpatient Care: Mixed Methods Study
Source: J Med Internet Res. 2024 Oct 18;26:e60306. doi: 10.2196/60306 (PMC11530726; doi:10.2196/60306)
Supplement: Multimedia Appendix 2 [file jmir_v26i1e60306_app2.docx]

# **Appendix 2. Survey.**

In January 2023, a collective survey, named EMC23, was digitally distributed to all 194 clinicians across the 35 subdepartments that collected PROMs data from specialty outpatients in 2022. The survey encompassed 26 questions for this study. Items and responses are presented in Table 1. Table 2 and Table 3 show the most prominent factors driving and constraining clinicians’ PROMs use.

**Table 1**. Survey items and responses.

| Item | Answer categories | N | % | |
| --- | --- | --- | --- | --- |
| 1. Sex | Male | 47 | 26 | |
|  | Female |  | 74 | |
|  | Other |  | 0 | |
| 1. Age | Average | 47 | 46 years | |
|  | Max |  | 64 years | |
|  | Min |  | 31 years | |
|  | StdDev |  | 8,3 years | |
| 1. Function | Medical Specialist | 47 | 64 | |
|  | Doctor in training |  | 4 | |
|  | Nurse specialist |  | 11 | |
|  | Nurse |  | 9 | |
|  | Nurse consultant |  | 6 | |
|  | Psychologist |  | 4 | |
|  | Sexologist |  | 2 | |
| 1. How often do you see the majority of your patients? | This varies significantly | 47 | 34 | |
|  | Multiple times over an extended period |  | 51 | |
|  | A few times in a short timeframe |  | 11 | |
|  | One time only |  | 4 | |
| 1. Average work hours per week | 17 - 32 hours | 47 | 15 | |
|  | 33 - 40 hours |  | 32 | |
|  | 41 hours or more |  | 53 | |
| 1. Years of work experience (excluding main education) | 2 - 5 years | 47 | 4 | |
|  | 6 - 10 years |  | 9 | |
|  | 11 - 25 years |  | 62 | |
|  | More than 25 years |  | 26 | |
| 1. When were PROMs first collected among patients in your department? | Between 2013 to 2019 | 47 | 38 | |
|  | Between 2020 and 2023 |  | 34 | |
|  | I don't know |  | 28 | |
| 1. Which PROMs are collected from your patients? | Generic PROMs and/or domain-specific PROMs | 45 | 29 | |
|  | Disease-specific PROMs |  | 16 | |
|  | Both generic/domain specific and disease specific PROMs |  | 38 | |
|  | I don't know |  | 18 | |
| 1. Were you present during the timeframe that your department started using PROMs? | Yes | 47 | 77 | |
|  | No, I started working there later |  | 13 | |
|  | I don’t know |  | 11 | |
| 1. How frequently do you examine patients’ responses to PROMs? | I have never used PROMs | 47 | 11 | |
|  | I have stopped looking at PROMs |  | 15 | |
|  | Occasionally |  | 36 | |
|  | As often as possible |  | 21 | |
|  | Always |  | 17 | |
| 1. *If in the previous question answer 3,4 or 5 was selected:*   To what extent do you discuss these outcomes with the patient during the consultation? | Never | 35 | 17 | |
|  | Occasionally |  | 29 | |
|  | As often as possible |  | 26 | |
|  | Always |  | 29 | |
| 1. *Among those that used PROMs at least once:*   What is/was/are/were the primary reason(s) for you to use PROMs in the consultation room? Multiple answers are possible.  (see also Table 2) | It benefits my patients | 42 | 38 | |
|  | It benefits me personally |  | 36 | |
|  | It is expected of me |  | 45 | |
|  | My patients request it |  | 12 | |
|  |  |  |  | |
|  | **Combinations with >5% occurance**  The combination "It benefits my patients" and "It benefits me personally" |  | 29 | |
|  | The combination “It benefits my patients”, “it benefits me personally”, and “it is expected of me”. |  | 7 | |
|  | Only: This is expected of me |  | 31 | |
|  | Only: My patients request it |  | 7 | |
| 1. *Among those that used PROMs at least once:* It is supportive that the PROM completion rate is shown at the frontpage of the Electronic Health Record. | Disagree | 42 | 17 | 17 |
|  | Partly disagree |  | 0 |  |
|  | Neutral |  | 19 | |
|  | Partly agree |  | 19 | 36 |
|  | Agree |  | 17 |  |
|  | No opinion |  | 29 | |
| 1. *Among those that used PROMs at least once:* The template to report   PROM results in a letter to another healthcare professional or general practitioner is supportive. | Disagree | 42 | 12 | 12 |
|  | Partly disagree |  | 0 |  |
|  | Neutral |  | 7 | |
|  | Partly agree |  | 0 | 2 |
|  | Agree |  | 2 |  |
|  | No opinion |  | 79 | |
| 1. What are the prominent reasons that you have possibly not been able to use PROMs optimally in the consultation room? Multiple answers possible.   (see also Table 3) | Dashboard functioning issues | 44 | 55 | |
|  | Not enough time |  | 55 | |
|  | Not in my routine, I forget it |  | 50 | |
|  | Misalignment with how I prefer to work |  | 27 | |
|  | Low volume of completed PROMs |  | 39 | |
|  | No added benefits for patients or myself |  | 25 | |
|  | Finding it difficult or perceiving not to possess necessary skills |  | 18 | |
|  | PROMs do not fit my patient population |  | 16 | |
|  | I have limited influence on improving PROM domains |  | 9 | |
|  | A colleague discusses PROMs |  | 5 | |
|  | Too little reinforcement |  | 5 | |
| 1. Al in all, what grade (1-10) do you give to the process of PROMs implementation in your consultation room?   (see also Table 3) | Average | 44 | 5,4 | |
|  | Min |  | 1 | |
|  | Max |  | 9 | |
|  | StdDvt |  | 2,4 | |
| 1. Al in all, what grade (1-10) do you give the outcomes of implementing PROMs in your consultation room?   (see also Table 3) | Average | 44 | 4,9 | |
|  | Min |  | 1 | |
|  | Max |  | 9 | |
|  | StdDvt |  | 2,3 | |
| 1. I received sufficient education to use PROMs in the consultation room. | Disagree | 43 | 30 | 56 |
|  | Partly disagree |  | 26 |  |
|  | Neutral |  | 26 | |
|  | Partly agree |  | 16 | 16 |
|  | Agree |  | 0 |  |
|  | No opinion |  | 2 | |
| 1. I received sufficient on-the-job coaching to use PROMs in the consultation room. | Disagree | 43 | 33 | 60 |
|  | Partly disagree |  | 28 |  |
|  | Neutral |  | 26 | |
|  | Partly agree |  | 12 | 12 |
|  | Agree |  | 0 |  |
|  | No opinion |  | 2 | |
| 1. Stories of other healthcare professionals influenced my attitude towards working with PROMs positively. | Disagree | 43 | 35 | 49 |
|  | Partly disagree |  | 14 |  |
|  | Neutral |  | 33 | |
|  | Partly agree |  | 16 | 16 |
|  | Agree |  | 0 |  |
|  | No opinion |  | 2 | |
| 1. The program team...   ...is helpful in the implementation of PROMs in my workplace. | Disagree | 43 | 14 | 23 |
|  | Partly disagree |  | 9 |  |
|  | Neutral |  | 40 | |
|  | Partly agree |  | 23 | 35 |
|  | Agree |  | 12 |  |
|  | No opinion |  | 2 | |
| 1. The program team ...   … communicates professionally and reaches me through appropriate channels. | Disagree | 43 | 12 | 19 |
|  | Partly disagree |  | 7 |  |
|  | Neutral |  | 37 | |
|  | Partly agree |  | 30 | 42 |
|  | Agree |  | 12 |  |
|  | No opinion |  | 2 | |
| 1. The program team ...   … provides sufficient customization for my team/department. | Disagree | 43 | 16 | 28 |
|  | Partly disagree |  | 12 |  |
|  | Neutral |  | 49 | |
|  | Partly agree |  | 16 | 21 |
|  | Agree |  | 5 |  |
|  | No opinion |  | 2 | |
| 1. The program team ...   … asks about our experiences with working with PROMs and the implementation process. | Disagree | 43 | 14 | 30 |
|  | Partly disagree |  | 16 |  |
|  | Neutral |  | 40 | |
|  | Partly agree |  | 23 | 28 |
|  | Agree |  | 5 |  |
|  | No opinion |  | 2 | |
| 1. The program team ...   … regularly provides feedback on the use of PROMs in my department. | Disagree | 43 | 21 | 47 |
|  | Partly disagree |  | 26 |  |
|  | Neutral |  | 35 | |
|  | Partly agree |  | 16 | 16 |
|  | Agree |  | 0 |  |
|  | No opinion |  | 2 | |
| 1. The program team...   … effectively encourages me to use PROMs in the consultation room. | Disagree | 43 | 19 | 47 |
|  | Partly disagree |  | 28 |  |
|  | Neutral |  | 37 | |
|  | Partly agree |  | 12 | 14 |
|  | Agree |  | 2 |  |
|  | No opinion |  | 2 | |

Note: Majority, i.e. 50% or more of respondents selected this answer, is color coded green.

**Table 2**. Prominent reasons for clinicians to use PROMs, both in total and per sub-group.

|  |  | **All respondents that used PROMs at least once (n=42)** | **Never attempters (n=5)** | **Ceased user (n=7)** | **Occasional users (n=17)** | **As often as possible users (n=10)** | **Always users (n=8)** |
| --- | --- | --- | --- | --- | --- | --- | --- |
|  |  |  |  |  |  |  |  |
| **Item** | **Answers** |  |  |  |  |  |  |
| Most prominent factors reasons why I use(d) PROMs | Benefits for the patient | 38% | Not applicable | 0% | 29% | 50% | 75% |
|  | Benefits for myself | 36% | Not applicable | 0% | 29% | 40% | 75% |
|  | It is expected from me | 45% | Not applicable | 43% | 53% | 40% | 38% |
|  | Patients requests | 12% | Not applicable | 29% | 18% | 0% | 0% |
|  | Other, namely… | 0% | Not applicable | 0% | 0% | 0% | 0% |
| Calculation | Average # of factors selected | 1,3 | Not applicable | 0,7 | 1,3 | 1,3 | 1,9 |

**Table 3.** Prominent factors that contributed to suboptimal use of PROMs, if experienced, both in total and per sub-group.

|  |  | **All respondents (n=44*)** | **Never attempters (n=4*)** | **Ceased user (n=7)** | **Occasional users (n=15*)** | **As often as possible users (n=10)** | **Always users (n=8)** |
| --- | --- | --- | --- | --- | --- | --- | --- |
|  |  |  |  |  |  |  |  |
| **Item** | **Answers** |  |  |  |  |  |  |
| Most prominent factors causing sub-optimal use of PROMs, if experienced | Dashboard functioning issues | 55% | 50% | 86% | 53% | 60% | 25% |
|  | Not enough time | 55% | 100% | 100% | 53% | 10% | 50% |
|  | Not in my routine, I forget it | 50% | 75% | 71% | 67% | 40% | 0% |
|  | Misalignment with how I prefer to work | 27% | 75% | 43% | 33% | 0% | 13% |
|  | Low volume of completed PROMs | 39% | 25% | 43% | 33% | 50% | 38% |
|  | No added benefits for patients or myself | 25% | 0% | 43% | 33% | 20% | 13% |
|  | Finding it difficult or perceiving not to possess necessary skills | 18% | 50% | 14% | 27% | 10% | 0% |
|  | PROMs do not fit my patient population | 16% | 25% | 29% | 13% | 0% | 25% |
|  | I have limited influence on improving PROM domains | 9% | 0% | 0% | 20% | 10% | 0% |
|  | A colleague discusses PROMs | 5% | 0% | 0% | 13% | 0% | 0% |
|  | Too little reinforcement | 5% | 25% | 14% | 0% | 0% | 0% |
|  | Other, namely… | 0% | 0% | 0% | 0% | 0% | 0% |
| Calculation | Average # of factors selected | 3,0 | 4,3 | 4,4 | 3,5 | 2,0 | 1,6 |
| Average grade from 1-10 | PROMs implementation process | 5,4  (min 1, max 9) | 3,3  (min 1, max 6) | 2,4  (min 1, max 4) | 5,3  (min 3, max 8) | 6,5  (min 4, max 9) | 7,8  (min 5, max 9) |
|  | PROMs implementation outcomes | 4,9  (min 1, max 9) | 3,3  (min 1, max 6) | 2,4  (min 1, max 5) | 4,9  (min 2, max 8) | 5,2  (min 3, max 9) | 7,5  (min 5, max 9) |
