# Supplementary material for: Enhancing Clinicians’ Use of Electronic Patient-Reported Outcome Measures in Outpatient Care: Mixed Methods Study
Source: J Med Internet Res. 2024 Oct 18;26:e60306. doi: 10.2196/60306 (PMC11530726; doi:10.2196/60306)
Supplement: Multimedia Appendix 3 [file jmir_v26i1e60306_app3.docx]

**Appendix 3. Semi-structured interview questions**

This interview guide was used to interview individuals involved in the hospital-wide transition to Value-Based Healthcare (VBHC), particularly focusing on the implementation of Patient Reported Outcome Measures (PROMs). After re-analysis, the following questions were found to effectively prompt relevant information concerning clinicians’ underuse of PROMs, hereafter issue x, and related strategies.

Questions that prompted insightful comments:

1. How did the hospital approach the implementation of PROMs as part of VBHC, and what specific actions did they undertake? What were the reasons behind these actions?
2. What were the primary challenges faced by the hospital during the implementation of PROMs? Probes (if necessary): can you elaborate on issue x. What caused issue x? What do you base your thoughts on? Can you think of other reasons/forces?
3. How did you or the hospital address issue x (response to Question 2)? Why was this approach chosen? Probes (if necessary): Please elaborate on this. What factors led to the selection of this particular approach/strategy/solution? What facilitated its implementation, and what were the main obstacles?
4. Have there been any modifications made to this approach/strategy/solution over time?
5. How do you reflect upon approach/strategy/solution X in addressing issue x? What was the outcome of employing this approach/strategy/solution regarding the issue? What factors contributed to these outcomes?
6. Were alternative responses considered for addressing the issue? Why were alternatives A, B, or C not pursued? What led to prioritizing approach/strategy/solution X?
7. In retrospect, what insights have you gained about the issue and its management? What changes would you propose for handling this issue differently in the future? Why? Probes (if necessary): What advice would you offer to other healthcare centers facing a similar issue? What resources or conditions are essential for resolving this issue optimally?
8. Is there anything that we have missed that you think is important to add? Please tell me.

General probes:

- Could you elaborate on that/tell me more about it?
- Could you provide an example?
- Is there anything else you would like to add?
- Are you suggesting that...?
- Does that imply/mean...?
- If I summarize this as..., would my understanding/interpretation be accurate?
