# Supplementary material for: Enhancing Clinicians’ Use of Electronic Patient-Reported Outcome Measures in Outpatient Care: Mixed Methods Study
Source: J Med Internet Res. 2024 Oct 18;26:e60306. doi: 10.2196/60306 (PMC11530726; doi:10.2196/60306)
Supplement: Multimedia Appendix 4 [file jmir_v26i1e60306_app4.docx]

**Appendix 4 . Specification of strategies**

Below, we specify each of the hospital's applied strategies. For 'implementation outcome(s) affected,' we note that the hospital did not specify these. In retrospect, we report on the most logical outcomes using CFIR’s Outcome Addendum (adoption/implementation/sustainment), except otherwise noted.

Capability-associated strategies

| **Domain** | **Strategy: kick-off and manual** |
| --- | --- |
| Actor(s) | The Central Support Team (CST) |
| Action(s) | A: Conducted a kick-off session  B: Shared a self-developed written manual via email, later supplemented with video instructions |
| Target(s) of the action | A and B: Clinicians who began to inquire Patient-Reported Outcome Measures (PROMs) among their outpatients, a decision made at the subdepartmental level |
|  | A and B: Capability - Awareness and knowledge about PROMs, skills to navigate the PROMs dashboard, information on how to contact the CST for further questions |
| Temporality | Often within one week following the initial inquiry (technical installation) of PROMs among outpatients |
| Dose | A: Conducted once, often during a dedicated time slot in a department meeting.  B: Distributed once |
| Implementation outcome(s) affected | Implementation |
| Justification | A: Logic – Boosts awareness and knowledge. Conducting the session during a department meeting ensures the highest likelihood of reaching as many clinicians as possible.  B: Logic – Enhances skills in navigating the PROMs dashboard. Email distribution ensures that all clinicians are reached. |

| **Domain** | **Strategy: pocket guide** |
| --- | --- |
| Actor(s) | The CST with input from clinicians |
| Action(s) | Developed and distributed a pocket guide on discussing PROMs with patients, available in print and digital |
| Target(s) of the action | Clinicians who inquired PROMs among their outpatients |
|  | Capability – practical examples of how PROMs could be discussed. |
| Temporality | Developed around 2022 |
| Dose | Print upon request, continuously available online |
| Implementation outcome(s) affected | Implementation |
| Justification | Logic - Practical examples from peers offer inspiration and guidance on how PROMs can be effectively discussed with patients. |

| **Domain** | **Strategy: Training sessions** |
| --- | --- |
| Actor(s) | The CST, often in collaboration with person-centered care group. |
| Action(s) | A: Developed and conducted trainings (e-modules and in-person training sessions) on PROMs use in practice.  B: Highlighted external training opportunities. |
| Target(s) of the action | A and B: Clinicians who inquired PROMs among their outpatients |
|  | Capability - Skills to discuss PROMs |
| Temporality | A and B: After technical installation/ initiation of PROMs |
| Dose | A and B: Offered with varying frequency and durations |
| Implementation outcome(s) affected | Implementation |
| Justification | A and B: Empirical evidence - Evaluations have shown that clinicians seek training on how to discuss PROMs, particularly for Shared Decision Making and when dealing with patients who have limited literacy. |

| **Domain** | **Strategy: Enabling trainigs** |
| --- | --- |
| Actor(s) | The CST |
| Action(s) | A: Made training flexible by offering e-trainings  B: Sought to provide accreditation for trainings |
| Target(s) of the action | Direct: educational context  Indirect: clinicians who want to attend training but face limited opportunities to do so |
|  | Opportunity for enhancing capability- access to trainings |
| Temporality | A: Ongoing from around 2018  B: Around 2022 |
| Dose | - |
| Implementation outcome(s) affected | Implementation |
| Justification | Empirical evidence - Observations indicated that clinicians were unable to attend training due to time constraints. |

| **Domain** | **Strategy: Central assistance** |
| --- | --- |
| Actor(s) | The CST |
| Action(s) | Provided accessibility for questions, addressed needs, offered training upon request, and proactively reached out to (sub)departments with low PROMs usage. |
| Target(s) of the action | All clinicians |
|  | Capability – addressing needs |
| Temporality | Ongoing |
| Dose | - |
| Implementation outcome(s) affected | Adoption, implementation, maintenance |
| Justification | Logic - The CST believed that adopting a personal approach and being easily accessible was crucial for effective support. |

| **Domain** | **Strategy: coaching on-the-job** |
| --- | --- |
| Actor(s) | The CST |
| Action(s) | Provided on-the-job coaching |
| Target(s) of the action | Clinicians seeking coaching on discussing PROMs during outpatient visits |
|  | Capability – knowledge and experience on how to use the PROMs dashboard and the practical discussion of PROMs |
| Temporality | After PROMs initiation / technical installation |
| Dose | A few outpatient consultations per person; available on request. |
| Implementation outcome(s) affected | Implementation |
| Justification | Empirical evidence - Local department experiences have shown this strategy to be effective. |

| **Domain** | **Strategy: peer-to-peer discussion** |
| --- | --- |
| Actor(s) | The CST |
| Action(s) | Organized peer-to-peer discussion events |
| Target(s) of the action | All clinicians |
|  | Capability - Knowledge about PROMs, skills to use PROMs, optimism about PROMs |
| Temporality | Since around 2021 |
| Dose | A couple of times each year, lasting a few hours each session. |
| Implementation outcome(s) affected | Adoption, implementation, sustainment |
| Justification | Logic and empirical evidence - Based on the belief and observed outcomes that clinicians can most effectively support their peers in understanding and utilizing PROMs, reinforced by observations. |

Opportunity-associated strategies

| **Domain** | **Strategy: hospital-wide awareness** |
| --- | --- |
| Actor(s) | The CST (including communication advisors, with occasional support from the communication department |
| Action(s) | A: Used hospital-wide channels and marketing to create hospital-wide awareness about PROMs and VBHC  B: Aimed to extend information provision to extramural parties, such as general practitioners |
| Target(s) of the action | A: All hospital staff, especially clinicians  B: General practitioners and care chain partners |
|  | Opportunity - awareness about PROMs and their central role in the hospital, insight in consequences for their work |
| Temporality | A: Ongoing  B: Planned |
| Dose | A: Varied  B: Not yet implemented |
| Implementation outcome(s) affected | A: Adoption, implementation, sustainment |
| Justification | A: Empirical evidence - Previous experiences have shown that without social support, the adoption and utilization of PROMs can be hindered.  B: Empirical evidence - Based on clinician feedback (evaluation 2020) |

| **Domain** | **Strategy: PROMs dashboard integrated in the Electronic Health Record (EHR)** |
| --- | --- |
| Actor(s) | The CST |
| Action(s) | A: Visualized PROMs in a dashboard  B: Integrated this dashboard in the Electronic Health Record (EHR) |
| Target(s) of the action | A and B: Direct: IT context  A and B: indirect: Clinicians who inquire PROMs among their outpatients |
|  | Opportunity - Enhancing ease of access and interpretation of PROM responses |
| Temporality | From the outset of clinicians' use of PROMs. |
| Dose | A and B: Ongoing |
| Implementation outcome(s) affected | A and B: Adoption, implementation, maintenance |
| Justification | A and B: Logic - It is believed that visualizing PROMs makes their use easier for clinicians, integrating them more seamlessly into their workflow.  B: Empirical evidence – Previous experience with a standalone dashboard highlighted limitations, motivating the shift towards integration within the EHR for improved functionality and accessibility |

| **Domain** | **Strategy: time saving strategies** |
| --- | --- |
| Actor(s) | The CST |
| Action(s) | A: Facilitated quick actions on PROMs outcomes  B: Streamlined other care processes |
| Target(s) of the action | A and B: direct: IT and care processes  A and B: indirect: clinicians who inquire PROMs among their outpatients |
|  | Opportunity – (perceiving) sufficient time to consider PROMs |
| Temporality | A and B: since around 2021, improvements ongoing |
| Dose | ongoing |
| Implementation outcome(s) affected | Adoption, implementation, sustainment |
| Justification | Logic - Ensuring that the use of PROMs is straightforward and time-efficient for clinicians is essential for adoption and sustainability.  Empirical evidence – identified time constraints in evaluations |

| **Domain** | **Strategy: PROMs completion status bar as reminder** |
| --- | --- |
| Actor(s) | The CST |
| Action(s) | Implemented a reminder to PROMs in the electronic health record (EHR) displaying patients' PROMs completion status using a status bar on the front page of their EHR. |
| Target(s) of the action | A and B: direct: IT context  A and B: indirect: clinicians who inquire PROMs among their outpatients |
|  | Opportunity – Enhancing memory and prompting clinicians to discuss PROMs. |
| Temporality | As of around 2021 |
| Dose | Ongoing |
| Implementation outcome(s) affected | Implementation, maintenance |
| Justification | Empirical evidence: Identified issues with remembering to discuss PROMs have highlighted the need for reminders.  Logic: Visibility of PROMs is crucial for ensuring that clinicians are reminded and prompted to engage in discussions about PROMs during patient interactions. |

| **Domain** | **Strategy: patient initiative** |
| --- | --- |
| Actor(s) | The CST |
| Action(s) | A: Facilitated patients to take the initiative to discuss PROMs by providing them with information on discussing PROMs  B: Upcoming: developing a dashboard to review their own scores |
| Target(s) of the action | A and B: Direct: patients that complete PROMs  A and B Indirectly: clinicians |
|  | Opportunity: Creating social influence that encourages and enables clinicians to use PROMs. |
| Temporality | A: With invitation to complete PROM  B: In development |
| Dose | A: once for every PROMs completion cycle  B: - |
| Implementation outcome(s) affected | Implementation and maintenance |
| Justification | A and B: Logic: Enabling patients to view their PROMs outcomes and encouraging them to discuss these with clinicians is expected to prompt discussions and increase engagement in PROMs use. |

Motivation-associated strategies

| **Domain** | **Strategy: involve executive board for commitment** |
| --- | --- |
| Actor(s) | The executive board |
| Action(s) | A: Verbally expressed commitment to achieve VBHC  B: Included VBHC as hospital aim |
| Target(s) of the action | A: All hospital staff  B: Policy context |
|  | Knowledge and tension for change |
| Temporality | A: Ongoing  B: Ongoing |
| Dose | A: A couple of times yearly  B: - |
| Implementation outcome(s) affected | Adoption, maintenance |
| Justification | Logic: Leveraging perceived urgency and creating tension for change, along with the influence of social commitment, can positively contribute to adoption and long-term maintenance of VBHC initiatives within the hospital. |

| **Domain** | **Strategy: involve executive board for monitoring progress and complimenting departments that excel** |
| --- | --- |
| Actor(s) | The executive board |
| Action(s) | A: Monitored departments’ implementation status  B: Complimented departments excelling in VBHC |
| Target(s) of the action | A: All subdepartments  B: Subdepartments excelling in VBHC |
|  | Reinforcement; policy prerequisites and compliments |
| Temporality | A and B: since around 2022 |
| Dose | A and B: Three times a year |
| Implementation outcome(s) affected | Adoption, maintenance |
| Justification | Logic – Formal pressure and social influence from the executive board can encourage departments to adhere to VBHC principles |

| **Domain** | **Strategy: participation** |
| --- | --- |
| Actor(s) | The CST and steering committee members |
| Action(s) | Enabled and invited clinician representatives to participate in the steering committee. |
| Target(s) of the action | Direct: Clinician representatives  Indirect: all clinicians |
|  |  |
| Temporality | Physicians involved since 2018; nurses since 2024. |
| Dose | Ongoing |
| Implementation outcome(s) affected | Adoption, maintainance |
| Justification | Logic – Involving clinician representatives in the steering committee fosters greater buy-in and acceptance among their peers |

| **Domain** | **Strategy: communication about VBHC** |
| --- | --- |
| Actor(s) | The CST (including communication advisor) |
| Action(s) | Communicated about VBHC to achieve and sustain clinicians’ interest |
| Target(s) of the action | All clinicians |
|  | Knowledge, motivation |
| Temporality | Ongoing |
| Dose | Frequent (e.g. monthly newsletter) |
| Implementation outcome(s) affected | Adoption, implementation, maintainance |
| Justification | Logic – By consistently communicating about VBHC, the CST aims to create and sustain interest and engagement among clinicians. |

| **Domain** | **Strategy: tailored (persuasive) communication with subdepartments** |
| --- | --- |
| Actor(s) | The CST |
| Action(s) | Provided tailored education to each (sub)department on PROMs and VBHC, employing persuasive arguments, addressing concerns, and facilitating open discussions. |
| Target(s) of the action | Clinicians in (sub)departments who consider using PROMs |
|  | Knowledge, motivation |
| Temporality | Ongoing |
| Dose | Tailored to each subdepartment's needs, typically conducted once or twice as needed. |
| Implementation outcome(s) affected | Adoption |
| Justification | Logic – A personalized and tailored approach to communication is more effective in convincing clinicians of the benefits and importance of using PROMs and embracing VBHC principles. |

| **Domain** | **Strategy: establish evidence** |
| --- | --- |
| Actor(s) | The CST |
| Action(s) | Aimed to demonstrate the impact of PROMs, including through academic partnerships |
| Target(s) of the action | Direct: research context  Indirect: all clinicians |
|  | Motivation – evidence of benefits and personal consequences |
| Temporality | Mainly as of 2021 |
| Dose | Low intensity; occasional setbacks. |
| Implementation outcome(s) affected | Adoption, maintenance |
| Justification | Empirical evidence – Clinicians have expressed a need for evidence demonstrating the benefits and personal consequences of using PROMs. |

| **Domain** | **Strategy: triability** |
| --- | --- |
| Actor(s) | The CST |
| Action(s) | Enabled (sub)departments to try PROMs with a subset of patients |
| Target(s) of the action | (Sub)departments and their clinicians |
|  | Motivation; To assess feasibility and benefits before making a full adoption decision. |
| Temporality | Just prior to adoption decision |
| Dose |  |
| Implementation outcome(s) affected | Adoption  Unintended consequence: implementation and maintenance |
| Justification | Logic - Trialing PROMs at a small scale allows (sub)departments and clinicians to evaluate its effectiveness and feasibility in their specific context. This approach can mitigate risks and uncertainties, making it more acceptable for broader adoption by demonstrating initial benefits and addressing concerns before committing fully. |

| **Domain** | **Strategy: Feedback** |
| --- | --- |
| Actor(s) | The CST |
| Action(s) | Gave tailored feedback on (sub)departments/clinicians’ use of PROMs |
| Target(s) of the action | (sub)departments/clinicians |
|  | Knowledge on their PROMs use rates, motivation, social influence |
| Temporality | After PROMs initiation/installment |
| Dose | Tailored based on agreement with (sub)departments; frequency ranges from monthly to a few times yearly. |
| Implementation outcome(s) affected | Implementation, maintenance |
| Justification | Logic – Providing tailored feedback serves as a reminder and motivator for (sub)departments and clinicians to continue using PROMs effectively. It also fosters social comparison, encouraging improvements and maintaining engagement over time. |

| **Domain** | **Strategy: adaptablity** |
| --- | --- |
| Actor(s) | The CST |
| Action(s) | Enabled (sub)departments/clinicians to adapt PROMs and their uses to fit local needs and contexts. |
| Target(s) of the action | (sub)departments/clinicians |
|  | Motivation; encouraging ownership and autonomy in adapting PROMs to local contexts |
| Temporality | Starting around 2021, focusing on increasing adaptability over time. |
| Dose | - |
| Implementation outcome(s) affected | Implementation, maintenance |
| Justification | Logic - By promoting the appropriateness and local fit of PROMs innovations, and allowing stakeholders to shape their implementation, this strategy enhances perception of acceptance, ownership and autonomy |

| **Domain** | **Strategy: extending benefits** |
| --- | --- |
| Actor(s) | The CST |
| Action(s) | Extended the use cases of PROMs |
| Target(s) of the action | Direct: care processes  Indirect: clinicians |
|  | Motivation - Enhancing the perceived benefits of PROMs to increase cooperation among clinicians |
| Temporality | Increasing over the years |
| Dose | Ongoing |
| Implementation outcome(s) affected | Adoption, implementation, maintenance |
| Justification | Logic – Clinicians have diverse motivations and perceptions regarding the usefulness of PROMs. By expanding the applications and demonstrating additional benefits of PROMs in various care processes, the CST aims to enhance their appeal and relevance to clinicians. |

| **Domain** | **Strategy: clarify accountability in patient information** |
| --- | --- |
| Actor(s) | The CST |
| Action(s) | Adapted patient information to alleviate clinicians' concerns about accountability |
| Target(s) of the action | Direct: patient communication  Indirect: clinicians |
|  | Motivation: Addressing concerns that could hinder adoption of PROMs, such as ensuring timely detection of critical patient information, like indications of suicidality reported in PROMs, by clinicians. |
| Temporality | As of around 2022 |
| Dose | Ongoing |
| Implementation outcome(s) affected | Adoption |
| Justification | Empirical evidence –Clinicians have cited concerns about accountability as a barrier to adopting PROMs. By adapting patient information to clarify how PROMs data is managed and the responsibilities involved, the CST aims to alleviate these concerns. This approach supports the adoption of PROMs by ensuring clinicians feel confident in using and acting upon patient-reported information without undue liability concerns. |

**Generic**

| **Domain** | **Strategy: conduct formal and informal evaluations and adjust plans** |
| --- | --- |
| Actor(s) | The CST |
| Action(s) | Conducted formal and informal evaluations to examine clinicians' experiences with PROMs, their wishes, and their needs. They used these insights to make necessary adjustments in the implementation plan. |
| Target(s) of the action | Direct: clinicians |
|  | Capability, Opportunity and/or Motivation: Identifying and addressing barriers, motivations, needs, wishes among clinicians using PROMs. |
| Temporality | As of 2020 |
| Dose | Formal evaluation annually, informal evaluations frequently |
| Implementation outcome(s) affected | Adoption, implementation, sustainment |
| Justification | Logic – the CST aimed to understand drivers of clinicians’ behaviors and facilitate their use of PROMs. |
